# “Purse-String” Capsular Closure for Decreasing Dislocation Rates in Proximal Femur Replacements

**DOI:** 10.5435/JAAOSGlobal-D-20-00086

**Published:** 2020-11-10

**Authors:** Thomas A. Novack, Jay N. Patel, Tyler Hoskins, Charles Long, Christopher Mazzei, David Goyette, James C. Wittig

**Affiliations:** From the Department of Orthopedic Surgery, Morristown Medical Center, Morristown (Dr. Novack, Dr. Patel, Mr. Hoskins, Dr. Long, Mr. Mazzei, Mr. Goyette, and Dr. Wittig), and the Department of Orthopedic Surgery, St. Joseph's Regional Medical Center, Paterson, NJ (Dr. Novack and Dr. Long).

## Abstract

Hip joint dislocation is the most common complication after a proximal femur replacement. As the utilization of proximal femur replacements continues to increase, it becomes imperative for surgeons to find the optimal method to decrease postoperative dislocation and its sequelae. These cases often involve extensive soft-tissue deficits that require reconstruction to provide postoperative strength and stability. Patients report good functional outcomes; however, dislocation remains a concern. Although “described” previously in the literature, the authors illustrate the “purse-string” hip joint capsular closure technique to help other surgeons understand it and apply to their practice as deemed necessary. We also present the senior author's results with using a modified version of the “purse-string” hip joint capsular closure technique.

Proximal femur replacements (PFRs) are generally reserved for patients with extensive bone loss in the oncologic and revision arthroplasty setting. Traditionally, orthopaedic oncology patients with primary sarcomas or extensive metastatic disease involving the proximal femur have undergone endoprosthetic reconstruction. The dislocation rate after modular endoprosthetic reconstruction in neoplastic cases has been reported as between 2% and 33%.^1,2,3,4^ In the revision arthroplasty setting, patients with failed total hip replacements (THA) and/or fracture fixation with poor bone stock or proximal bone loss have had dislocation rates between 10% and 25% after PFR.^5,6^ Although patients with PFRs report good functional outcomes,^7,8,9,10^ dislocation remains a concern for many patients undergoing this procedure.^11,12^ Dislocation in this setting can not only cause extensive structural damage (both to the surrounding soft-tissue support and to the components themselves) but may also result in additional surgeries and negative psychological ramifications for patients recovering from surgery.^13^ Although several studies have addressed the issue of dislocation with PFRs using different soft-tissue techniques, postoperative stability remains a concern.^1,3,4,9^

As the utilization of PFRs continues to increase, a pressing need exists to minimize the rate of dislocation. The authors illustrate the “purse-string” capsular closure technique with a modification for soft-tissue reconstruction in patients who underwent a PFR in the orthopaedic oncology setting. Although this technique has been described previously, much of the orthopaedic community in other subspecialties/general orthopaedics may not be familiar of its benefits. Our goal is to illustrate this technique to help other surgeons understand it and apply to their practice as deemed necessary. We also review the outcomes of the senior surgeon (J.C.W.) with this technique.

## Methods

A retrospective review was done using single-surgeon (J.C.W.) data for all patients who underwent a “purse-string” capsular reconstruction technique for capsular repair after undergoing a PFR. Inclusion criteria included patients undergoing a primary PFR using the “purse-string” capsular closure technique done between January 2010 and February 2018. Exclusion criteria included any patients who had a follow-up of less than 12 months. Functional outcomes were assessed using the Musculoskeletal Tumor Society (MSTS) scoring system for the lower extremity. Data analysis was performed using Microsoft Excel and the Fisher exact test. The results were deemed statistically notable if the calculated *P* value was <0.05. Specifically, we analyzed patient demographics, indications for PFR, and rate of postoperative.

## “Purse-String” Capsular Repair Technique

**Figure 1:**
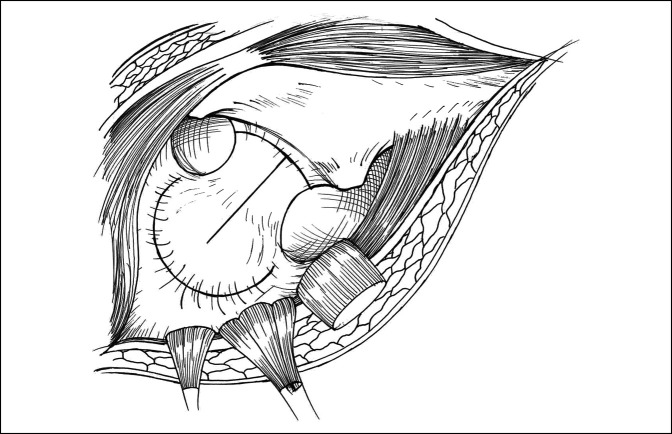
Illustration demonstrating the short external rotators released from their attachment on the femur and retracted posteriorly exposing underlying hip joint capsule. A T-shaped capsulotomy is made with the vertical limb going over the long axis of the femoral neck and the horizontal limb going parallel to the intertrochanteric line along the base of the femoral neck.

**Figure 2:**
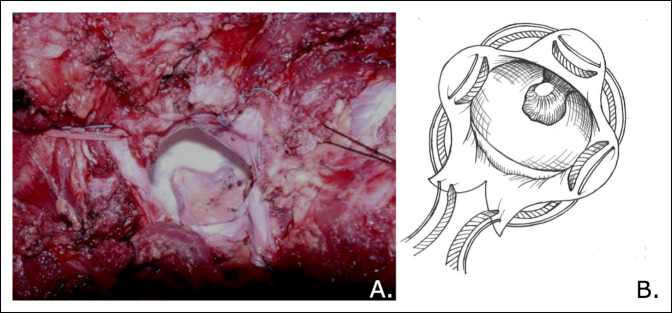
Photograph and illustration demonstrating the #5 FiberWire and 5 mm Merselene tape woven through the capsule in a purse string manner.

**Figure 3:**
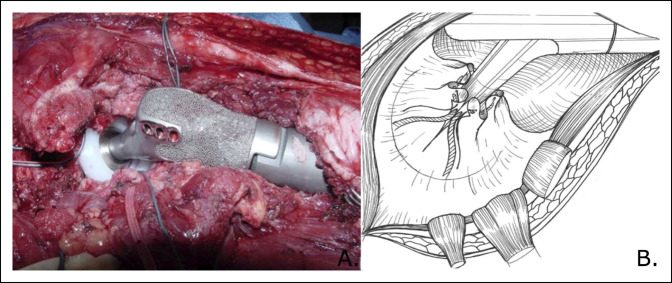
Once the endoprosthesis is reduced within the acetabulum (photograph), the #5 FiberWire and 5-mm Merselene tape are then cinched to tighten the noose around the prosthetic neck and tied to form a secure capsular repair (illustration).

**Figure 4:**
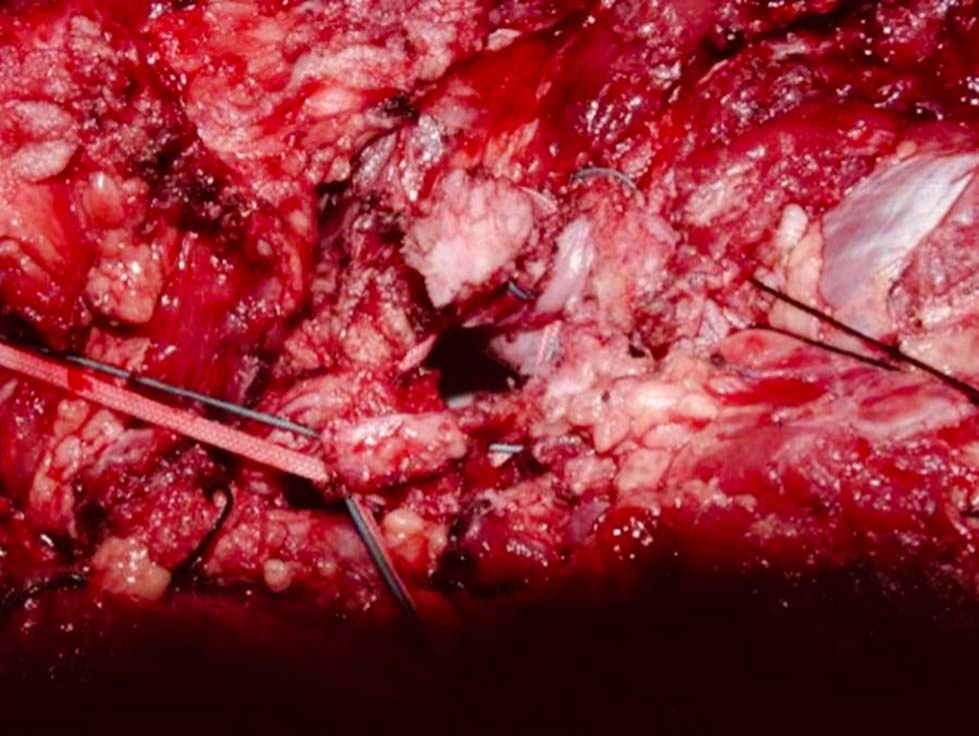
Intraoperative photograph showing the #5 FiberWire and Mersilene tape synched down to demonstrate how the capsule would close around the neck of the endoprosthesis.

An extensile posterolateral approach to the hip and proximal femur was done on all patients included in this study. First, the piriformis was released from its insertion, along with the other hip external rotators, and tagged with sutures. Subsequently, a “T-shaped” capsulotomy was created through the joint capsule, preserving as much capsular length as possible (Figure 1). The femur/tumor was then separated from the pertinent musculature during the resection using oncologic principles. During the resection, the iliopsoas was preserved if it not involved by tumor, separated from the anterior hip capsule, and tagged with sutures. After resecting the proximal femur and testing the stability with trial components, the hip joint capsule was prepared before positioning of the final implants. Two separate “purse-string” heavy nonabsorbable sutures were weaved through the capsule toward its perimeter, starting in the 6 o'clock position and moving clockwise to the 5 o'clock position, similar to a “purse-string.” A #5 FiberWire (Arthrex) was woven a few mm from the edge of the capsule (Figure 2). Next, a 5-mm Mersilene tape (Ethicon) was woven in a similar fashion approximately 0.5 cm proximal to the FiberWire. The PFR component was cemented into position during the reconstruction phase in the appropriate anteversion. Once the cement cured, the femoral prosthesis was reduced into the acetabulum. The two free ends of the #5 FiberWire were then tied together creating a noose around the femoral neck. Finally, the two free ends of the 5-mm Mersilene tape were tied together, reinforcing the noose created by the tied FiberWire ends (Figure 3). Figure 4 shows the #5 FiberWire and Mersilene tape synched down to demonstrate how the capsule would close around the neck of the endoprosthesis.

The iliopsoas tendon was rerouted and brought over the superior neck and sutured to the piriformis and external rotators using heavy nonabsorbable sutures. This creates a two-layer repair around the posterior/superior aspect of the prosthesis. The hip abductors were repaired to the proximal part of the prosthesis using heavy nonabsorbable sutures. In general, if the greater trochanter can be saved, a trochanteric claw mechanism may be used for the hip abductor repair. The hip was kept in 45° of abduction while repairing the soft tissues. The vastus lateralis muscle was used to cover most the prosthesis when it was repaired proximally to the prosthesis or to the abductor mechanism. In our series, the vastus lateralis was able to be preserved in all of the patients.

The acetabulum was not resurfaced, and all patients underwent a bipolar hemiarthroplasty. Postoperatively, all patients were treated with a hip abduction brace for 6 to 8 weeks and were made weight-bearing as tolerated. The hip abduction brace was set to 20° of hip abduction and allowed up to 60° of hip flexion. Posterior hip precautions were followed for 12 to 16 weeks. Hip abduction exercises were initiated at 6 weeks after surgery. If indicated, the patients were allowed to begin radiation therapy to the area once the skin incision had healed, which was approximately four to six weeks postoperatively.

## Results

Eleven patients who met the inclusion criteria were identified. The mean age of the patients was 57.8 years, and the mean body mass index was 24.53; there were 4 men and 6 women. The average follow-up was 34.1 months (range, 18-117). Six patients were treated for a primary lesion, and five patients were treated for metastatic disease. Their diagnoses included three patients with a chondrosarcoma, one with Ewing sarcoma, one with dedifferentiated chondrosarcoma, one with adenocarcinoma, one with pseudotumor, one with hemophilic pseudotumors, one case of metastatic prostate disease, and two patients with large cell lymphoma. Seven patients were treated with a Stryker proximal femur prosthesis and four with a Zimmer Biomet prosthesis. The mean length for each prosthesis was 20.8 cm (range, 17-28.3 cm).

The mean MSTS functional outcome score before surgery was 13.2 (range, 10-15), compared with 24.6 (range, 22-28) after surgery (*P* < 0.01). There were no dislocations reported in any of the patients at the time of latest follow-up (range, 14-113 months). Five patients required additional procedures to the area because of infection (2 patients) and hardware loosening (3 patients). Both infection cases involved only superficial wound drainage and occurred at a mean of 1.3 years (range, 12-19 months) after the index procedure in the 80- and 84-year-old patients, respectively. There were three cases of aseptic prosthetic loosening—two involving the greater trochanteric claw and one involving the distal femoral stem. They occurred at a mean of 4.5 years (range, 14-84 months) after the index procedure. There was one patient who presented with a local recurrence of a dedifferentiated chondrosarcoma 22 months after index procedure and ultimately underwent a hemipelvectomy.

## Discussion

One of the most common, and devastating, complications that can occur after PFR is postoperative dislocation. Previous literature has reported high dislocation rates in tumor patients who undergo modular endoprosthetic reconstruction. The technique described here has been modified in that we wove 2 “purse strings” around the capsule, a #5 FibeWire around the periphery, and a 5 mm Mersilene tape a about 0.5 cm more central. We first synch the peripheral #5 FiberWire, followed by the 5 mm Mersilene tape. This is done to give added stability and incase one of the “purse string” sutures fail either because of poor tissue or failure of the suture itself. With no dislocations at an average follow up of 34.1 months, our series of substantiates this evidence and helps further evaluate and validate the efficacy of a “purse string” capsular closure technique to reduce dislocation rates in PFRs.

Bickels et al^1^ first described the technique outlined in this study for their soft-tissue reconstruction technique for PFR in 39 patients who underwent a proximal femur resection because of a malignancy. Their technique emphasized preservation of the acetabulum and specifically reconstruction of the abductor mechanism to restore joint stability and avoid dislocation. For their capsular reconstruction, they weaved a single 3-mm Dacron tape (Deknatel), through the capsule, and synched it down around the neck of the prothesis. They described it as forming a “noose” around the neck of the endoprosthesis. Only one patient (1.7%) in their cohort experienced a postoperative dislocation. Henderson et al^14^ further expounded on the technique, using the term “purse string” capsular closure with excellent results. They used a single 3-mm Cottony Dacron suture and wove it through the capsule to close it like a “purse string” in patients who underwent a hemiarthroplasty because of neoplastic disease. Of the 36 patients included in their study cohort, only one patient experienced a postoperative dislocation (2.8%). Chiu et al^15^ used the “purse string” technique in 16 patients and reported no dislocations at a mean follow-up of 41.9 months. They used a 6-mm nylon tape in their earlier cases and then changed to Ethibond (Ethicon) for their later cases. They found the Ethibond was easier to handle in cases with a thinner capsular cuff.

Previous literature has reported high dislocation rates in tumor patients who undergo modular endoprosthetic reconstruction with various other capsular closure techniques. Puchner et al^3^ reported a 13% overall dislocation rate on 166 patients who underwent PFR between 1982 and 2008. Zehr et al^4^ reported dislocation rates as high as 18% in patients undergoing PFR with soft-tissue repair, with functional outcomes reported to be 80% based on the MSTS criteria. Similarly, Ilyas et al^2^ reported a 20% dislocation rate in 15 patients who underwent tumor salvage with a modular endoprosthesis with a mean follow-up of 6.7 years. Other authors have reported the outcomes of endoprosthetic reconstructions in a revision arthroplasty setting. Viste et al^6^ reported on 44 patients between 2000 and 2013 who underwent PFR for failed total hip arthroplasty, with a mean follow-up of 6 years. 6/44 (13.6%) patients suffered a dislocation event, at a mean of two years postoperatively. Al-Taki et al^13^ reported on 36 patients who underwent a PFR between 1996 and 2006 for non-neoplastic conditions, with an average follow-up of 3.2 years. Three of 36 patients (8.3%) in their series sustained at least one dislocation.

There were several limitations to our case series with this technique. All of the patients were tumor cases. In complex revision arthroplasty where the anatomy has been distorted by previous surgical intervention, the capsule itself may not be supple and may be scarred, making it difficult to use the described technique. There were only 11 patients included in this series; however, PFRs are an uncommon procedure.

By illustrating the “purse string” technique, the authors feel that it can be of help in a broader orthopaedic setting such as in PFRs, revision hip arthroplasty, and primary hip arthroplasty in patients at risk for dislocation.
